# Diffusion Mechanism of Rejuvenator and Its Effects on the Physical and Rheological Performance of Aged Asphalt Binder

**DOI:** 10.3390/ma12244130

**Published:** 2019-12-10

**Authors:** Fusong Wang, Lei Zhang, Boxiang Yan, Dezhi Kong, Yuanyuan Li, Shaopeng Wu

**Affiliations:** 1 State Key Laboratory of Silicate Materials for Architectures, Wuhan University of Technology, Wuhan 430070, China; wangfs@whut.edu.cn (F.W.); yanboxiang@whut.edu.cn (B.Y.); Kongdz@whut.edu.cn (D.K.); Liyuanyuan@whut.edu.cn (Y.L.); 2 Department of Civil and Environmental Engineering, Norwegian University of Science and Technology, 7491 Trondheim, Norway; lei.zhang@ntnu.no

**Keywords:** aged asphalt binder, rejuvenator, rheological property, diffusion, emulsified asphalt

## Abstract

Using rejuvenator to improve the asphalt pavement service state has become an increasing concern in recent years. This study mainly focuses on the diffusion analysis between rejuvenator and aged asphalt, and further rheological influences by addition of rejuvenators. First, two rejuvenators, oil rejuvenator (OR) and penetrative rejuvenator (PR), were prepared in the laboratory. Afterward, the diffused performance of rejuvenators was investigated by viscosity, contact angle, and three self-designed feasible test indexes, which were sinking time test, softening rate test, and gravitational collapsing test. Beside the comparison in physical properties tests of aged and rejuvenated asphalt, their rheological performances were also evaluated by dynamic shear rheometer (DSR) and bending beam rheometer (BBR) respectively. The results indicated that three proposed indexes can comparatively analyze the diffusion rate of different rejuvenators on aged asphalt effectively. Furthermore, all adopted test indexes signaled that PR has a faster diffusion rate and better penetrative efficiency. Comparatively, exceeding rejuvenator dosage would increase the rutting possibility. Nevertheless, 2.5 wt% addition dosage for both OR and PR into aged asphalt can promote appropriate improvement in physical properties and crack resistance. The study would supply a significant reference for penetrative rejuvenator preparation and its diffusing evaluation.

**Highlights**

- Two rejuvenators were prepared with waste cooking oil and emulsified asphalt;
- Three self-designed tests were used to study the diffusion efficiency of the rejuvenators;
- Dosages of rejuvenators were optimized based on the rheological property of asphalt;
- Two rejuvenators improved the crack resistance of aged asphalt;
- Penetrative rejuvenator has a better permeability to penetrate in aged asphalt.

## 1. Introduction

With the palpable trend of environmental sustainability development in the field of asphalt pavement [1,2], the preventive maintenance technology has been given increasing emphasis which has proved as a promising approach for alleviating pavement distresses and prolonging service life [3]. Preventive maintenance technology can make a significant difference by using some preliminary methods to control the pavement distresses before deterioration occurs [4,5]. Otherwise, greater consumption of material resources will be required for pavement reconstruction.

Preventive maintenance methods which included micro-surfacing, slurry seal, fog seal, and chip seal have been used to protect asphalt pavement in the recent years [6,7]. Nevertheless, the fog seal method attracted the most attention with its convenience in the application process and short construction period [8]. It means that spraying fluid material, such as rejuvenator, emulsified asphalt, and modified asphalt, creating a protective film on the pavement which fills the road cracks, stabilizes loose aggregates and results in an extended life of the aged asphalt [9,10,11]. Hence, the fluid material is the key point of fog seal method to achieve efficient results.

Not only in the field of preventive maintenance, rejuvenators were also warmly discussed in remixing with reclaimed asphalt pavement to deal with solid waste and meet road performance again [12,13,14,15]. Martins [16] integrated 10 optional places of asphalt plant to add rejuvenator (distilled tall oil) for better rejuvenation effects, and conveyor belt of cold reclaimed asphalt pavement (RAP) was recommended to obtain the improvement in asphalt fatigue and crack propagation resistance. Ali [17] claimed that using rejuvenator (date seed oil) could lead to better fatigue life of RAP. Similarly, with high RAP contained, Lu [18] investigated commercial rejuvenators’ application in warm mix asphalt mixture, whose result showed that rejuvenators performed better than Evotherm in fatigue cracking resistance. Additionally, bio-oil from sawdust [19], pyrolyzed tire [20], cyclogen [21], petroleum derivatives [22], and waste cooking oil (WCO) [12] were utilized as rejuvenators in many researches.

In relation to the fog seal, Zhang [23] analyzed the application of rejuvenator in porous asphalt pavement, and found it can improve the bending stiffness and raveling resistance. Cui [24] explored the feasibility for using silicone resin polymer in fog seal maintenance. Feng [25] tested the practical application possibility by mixing various contents of sand with modified bio-oil. Wan [26] investigated the chemical structure and rheological properties of aged styrene-butadiene-styrene modified asphalt with two kinds of rejuvenator materials, and thought that high maltene content could significantly soften the aged binder. Utilizing a commercial rejuvenator, O’Connell [27] evaluated the guidelines and procedures of recycling asphalt with seal maintenances, as there was no standard available in South Africa then. Moreover, two patents associated with fog seal rejuvenation from the United States [28,29] were published in the same year.

However, good permeability is the important precondition for the high efficiency of the rejuvenator; the permeability and diffusivity of rejuvenator has been seldom researched before [30]. After spraying on the asphalt pavement, the maintenance effectiveness of rejuvenator mainly depends on the permeable depth and diffused area in asphalt mixture. Meanwhile, the experimental methods in assessing and quantifying rejuvenator’s permeability and diffusivity to asphalt binder also has not been comprehensively researched, which strongly restrict the further application of rejuvenator in the preventive maintenance field.

In addition, biomass energy is a renewable energy which has the characteristics of large reserves, wide distribution, and environmental protection. WCO, belonging to current biomass energy, has been researched to produce biodiesel and functional chemicals [31]. In this study, the WCO was added to the emulsified asphalt to prepare the oil rejuvenator (OR). Because, when the dosage of WCO is 7%, the penetration, softening point, and ductility of rejuvenated asphalts can be restored to more than 90% of the original asphalt. WCO can be used as the rejuvenator and make a difference [32].

The objective of this study is to develop a kind of penetrative rejuvenator (PR) in laboratory, and evaluate the effects of its application in aged asphalt. First, the emulsified asphalt, WCO, and mixed penetrants were processed as OR and PR. Then the regenerating performance of OR and PR were investigated, including physical properties (penetration test, ductility test, and softening point test) of asphalt, high temperature shearing, and low temperature creeping. Besides that, viscosity and contact angle were measured, three concise and feasible test indexes were proposed assessing the permeability and diffusivity of rejuvenators which were sinking time test, softening rate test, and gravitational collapsing test. From this study, the rejuvenating properties and permeability of the rejuvenators were evaluated, and three feasible test indexes were proposed which can make a good reference in later penetrative rejuvenators’ research.

## 2. Materials and Experiments

### 2.1. Raw Materials

The 90-grade heavy traffic asphalt binder was used as the raw material. Its fundamental properties are shown in Table 1. Lignin amine, which was synthesized via the Mannich reaction, was used as the cationic emulsifier to produce emulsified asphalt [33]. It was an effective approach to recycle the biopolymer and reduce pollution of black liquor [34]. WCO was use as the light component supplemental resource for rejuvenators in this study, and Table 2 presents its properties. In order to obtain the PR, composite penetrants which were produced by fatty alcohol polyoxymethylene ether and ethylan 1005 (nonionic surfactant based on a synthetic primary alcohol) were mixed with OR to decrease the interfacial tension [35].

### 2.2. Experiments

**Figure 1:**
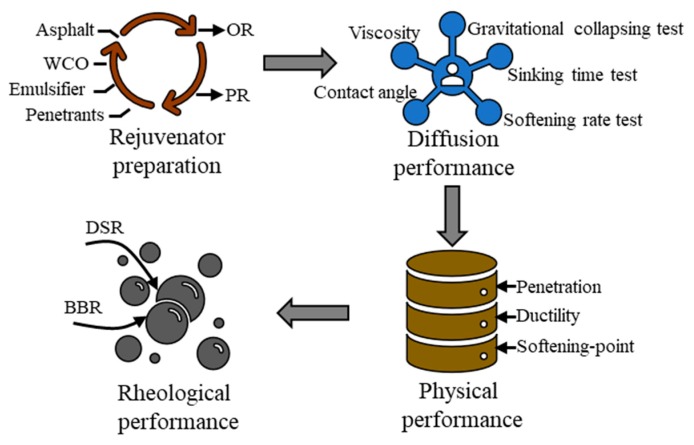
Schematic diagram for general research flow.

This study prepared two kinds of rejuvenators (OR and PR) with WCO and emulsified asphalt in the laboratory. In order to evaluate their feasibility in rejuvenating aged asphalt, penetrative analysis was first conducted with five tests. After that, the comparison of physical and rheological performances for rejuvenated and aged asphalt was studied. Accordingly, Figure 1 depicts the schematic diagram for general research flow.

#### 2.2.1. Preparation of Rejuvenators and Samples

**Figure 2:**
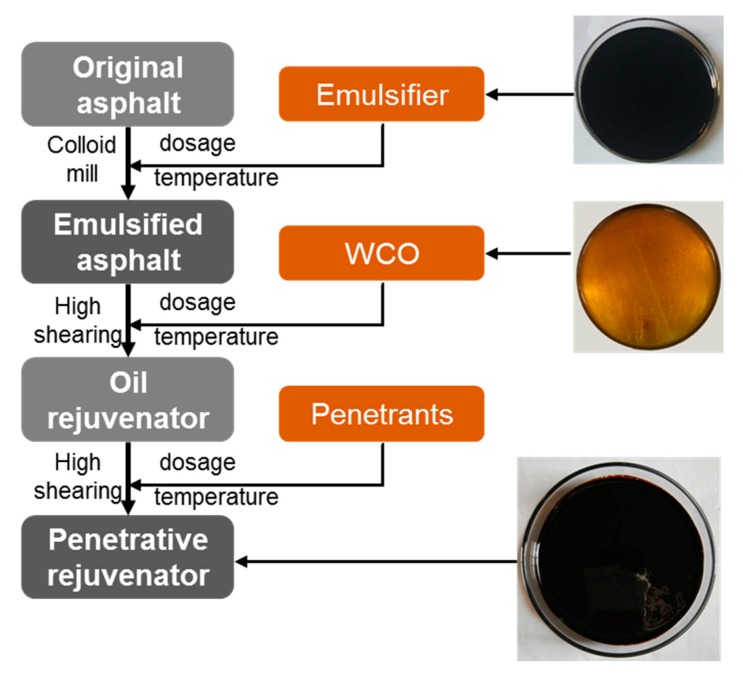
Flow chart for preparing oil rejuvenator (OR) and penetrative rejuvenator (PR).

Rejuvenators need to diffuse and penetrate into the aged asphalt mixtures during fog seal application, thereby refreshing the aged asphalt binder and repair micro cracks. Hence not only the regenerating components refreshing the aged binder is important, but the penetrative properties in the aged binder should be a point guaranteed [36]. Therefore, WCO was used as the regenerating components supplier, while emulsifier and penetrants were added to enhance the diffusivity and permeability [37,38]. First, a colloid mill (MIDE, Jiaxing, China) was used to prepare emulsified asphalt. Second, 40% mass ratio of WCO was added into the emulsified asphalt by a high speed shearing machine (5000 rpm, 120 min, ELE Mechanical & Electrical Equipment CO., LTD., Shanghai, China) to obtain OR. Third, 0.8% mass ratio of composite penetrants were introduced into the OR at 85 °C to produce PR (5000 rpm, 30 min). Figure 2 shows a brief preparation process of OR and PR.

Since fog seal maintenance was usually applied before the disease becomes evident, the study obtained the aged asphalt by treating with thin film oven test (TFOT, Huanan Experimental Instrument Co., Ltd., Wuxi, China) to simulate the situation. In this research, dosages of rejuvenator with 2.5 wt%, 5 wt%, and 10 wt% were studied via comparison with their rheological and diffused influence on aged asphalt binder. Before dropping rejuvenator into asphalt, the aged asphalt was heated until fluid at 100 °C. Then different amount of rejuvenators were mixed with aged asphalt to ensure it is diffusing uniformly. Besides original asphalt (Original-A) and aged asphalt (Aged-A), six samples were analyzed which are listed in Table 3.

#### 2.2.2. Diffused Performance Tests

Viscosity of the rejuvenators and the contact angle [39] between the aged binder and rejuvenators were first characterized. Then, three self-designed tests were proposed to evaluate the diffusion of the rejuvenator in aged asphalt binder, which were named as sinking time test, softening rate test, and gravitational collapsing test. These three tests provided a concise reference to characterize the diffusion of rejuvenators, by means of dissolving times.

**Figure 3:**
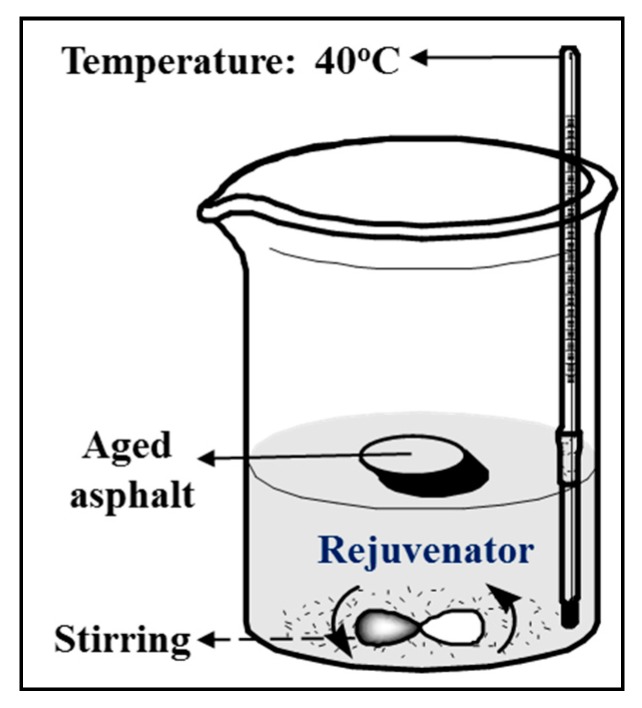
Schematic diagram of sinking time test.

**Figure 4:**
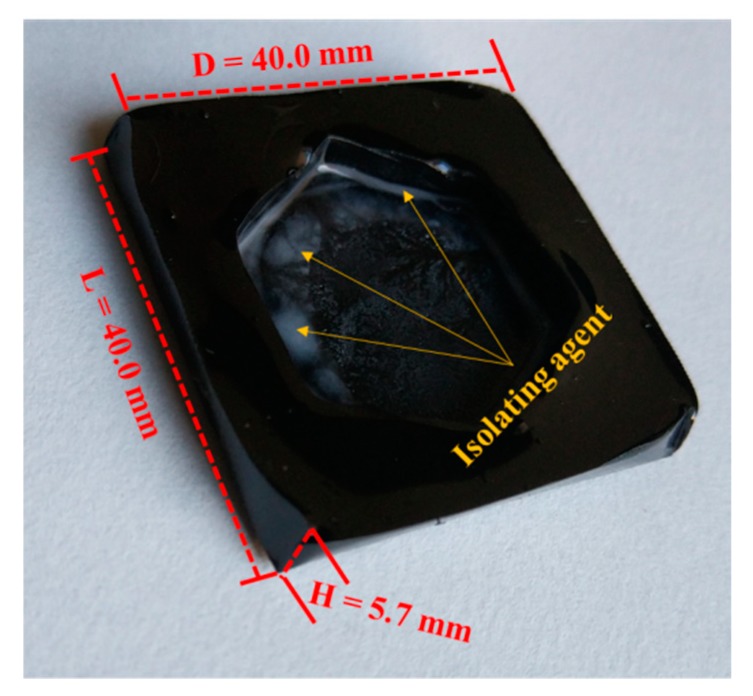
The size of aged asphalt in small sheet shape.

**(1) Sinking time test**. The aged asphalt specimen was kept floating on the rejuvenator. Stirring magnetic rotor was used in the whole testing process to keep the temperature, at 40 °C, in the rejuvenator uniform. The aged asphalt specimen was designed in a special shape that can ensure floating on the liquid rejuvenator. During the test, substance exchange occurs under the effects of molecular motion between the aged asphalt and liquid rejuvenator, so that the specimen will become soft and finally sink to the bottom. Sinking time to reach the bottom can be used to characterize the diffusion of the rejuvenators. Figure 3 indicates the schematic diagram. Figure 4 shows the size of the aged asphalt binder in small sheet shape of 40 mm × 40 mm × 5.7 mm.

**Figure 5:**
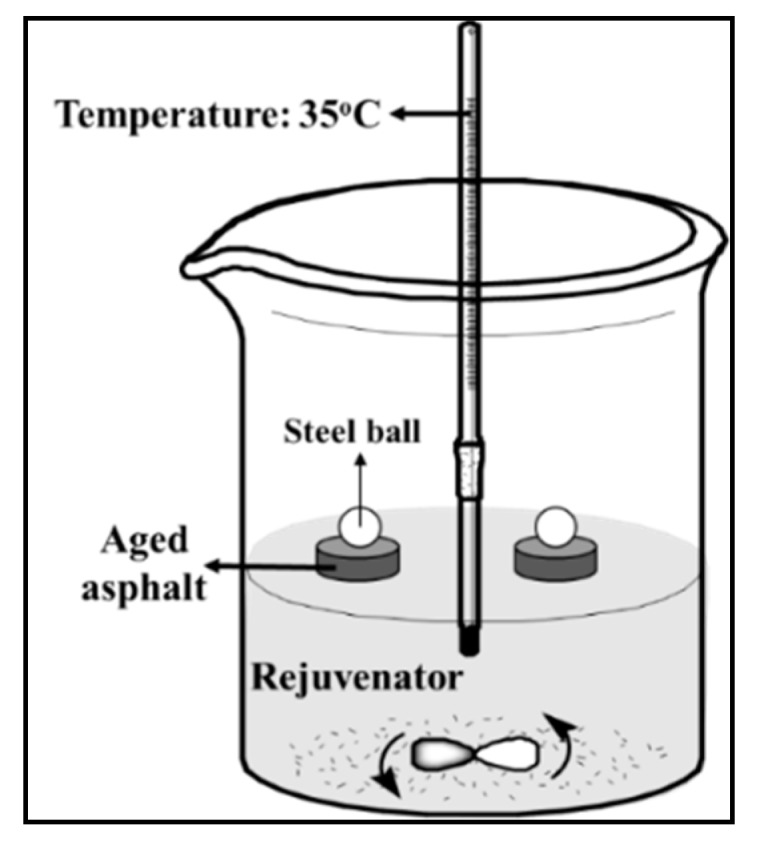
Schematic diagram of softening rate test.

**(2) Softening rate test**. It is a kind of revised softening-point test. Rejuvenator replaced water and was kept at a constant temperature. The test principle is to measure the time for aged asphalt to completely soften and stretch to touch the bottom under the gravity of a fixed steel ball. First, two specimens of aged asphalt were made by the softening-point test mold, and then placed softening point balls onto the specimens individually. Second, the specimens were placed in a beaker with the height of rejuvenator being 30 mm. Moreover, stirring magnetic rotor and water bath heating were used to keep the system temperature uniform at 35 °C. The time was recorded as the aged asphalt specimens touches the bottom. The schematic diagram is depicted in Figure 5.

**Figure 6:**
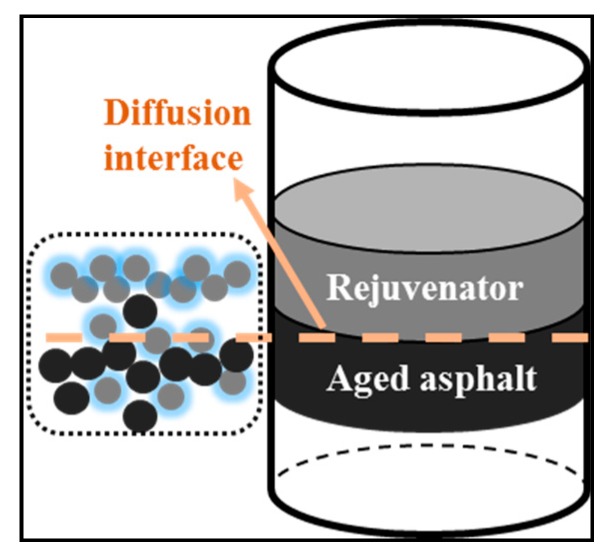
Schematic diagram of gravitational collapsing test.

**(3) Gravitational collapsing test.** It is designed to simulate the two phases diffusing rate at room temperature. The aged asphalt was dumped in a steel pipe whose another port is blocked up. Then rejuvenator was poured onto the solidified asphalt after it is cooled. At the two phases’ interface, rejuvenator and aged asphalt will diffuse to each other until the aged asphalt fully softens and crushes down. In this way, the crushing time of the aged asphalt under gravity can be used to analyze the rejuvenator’s diffusivity, as shown in Figure 6. The inner area of the mold is 4.23 cm^2^, and the used rejuvenators’ quality is 1.280 g. Hence, the pressure was 30.26 Pa on the diffusion interface. Moreover, the indoor temperature was 12 °C during the experiment.

#### 2.2.3. Rheological Performance Tests

First, the penetration test at 25 °C, ductility test at 5 °C, and softening-point test were conducted to investigate the samples’ physical properties. The rheological properties of aged asphalt always need to be considered with its brittleness at low temperature and rutting resistance at high temperature [40]. Then, dynamic shear rheometer (DSR, Anton Paar, Shanghai, China) and bending beam rheometer (BBR, Anton Paar, Shanghai, China) were used to evaluate the effect of two rejuvenators on the rheological properties of aged asphalt binder.

## 3. Results and Discussions

### 3.1. Diffusion Performance

Table 4 shows the results of diffusion tests for PR and OR, involved in viscosity, contact angle, and three self-proposed test methods. After the addition of penetrants, OR had an obvious decrease in viscosity, nearly 800 cP at room temperature. It reflected that the addition of composite penetrants allowed a smooth diffusion of the PR. Meanwhile, after dropping PR and OR onto the asphalt film samples, the results of the contact angle demonstrated that PR almost got a lesser contact angle than OR on both aged asphalt and original asphalt (approximate 20°). That illustrated that PR had a lower surface energy, which caused an obvious decrease in the interfacial tension between asphalt and rejuvenators. The addition of penetrants improved the efficiency in diffusion and permeability so that PR could spread out on the surface more easily. Compared to the original asphalt, the slight increase of contact angle for aged asphalt was explained as the aging process would increase the polarity, because nonpolar oil components are gradually transferred to polar resins and asphaltenes.

In the sinking time test, the aged asphalt specimen would soften and deform because of the effects of molecular exchange and buoyancy, thus sinking to the bottom. From the results of sinking time in Table 4, it was known that PR took 1557 s to immerse the sample, while OR lasted 2660 s and 17 min more than PR to diffuse and penetrate into the sample completely. It indicated that both PR and OR had softened the aged asphalt and caused gradual deformation generally. Moreover, PR had a better permeability into the aged asphalt. Therefore, PR could improve the efficiency in fog seal applications.

Softening rate test aims to measure the time as aged asphalt specimen stretch to constant length under the constant pressure and temperature conditions. OR lasted 1974 s to soften and stretch the aged asphalt, while PR was more efficient by 10 min ahead of time under the same condition. This means PR has a better permeability on the aged asphalt and diffuses to deeper parts for effective maintenance application.

As spraying on the asphalt pavement, the rejuvenator would contact and penetrate to the aged asphalt. Gravitational collapsing test simulated the penetration process of rejuvenator under normal conditions. With the effects of rejuvenators’ gravity and penetrating, suspended specimen would fall down as it softens. It was 1926 s for PR to penetrate and soften the aged asphalt, which was 16 min ahead of time than OR. It was concluded that PR could penetrate and diffuse to deeper parts under same condition, hence PR had a better permeability compared to OR.

### 3.2. Diffusion Mechanisms

Diffusion for rejuvenator penetrating into asphalt can be interpreted as the result of random molecular movements, which is called Brownian motions. Cussler [41] concluded that the significant factors in influencing the rejuvenator diffusing rate mainly involved in the size, shape of molecules or agglomerations, intermolecular forces, temperature, and so forth.

#### 3.2.1. Diffusion Theory

Fick’s law [42,43] and Stoke–Einstein equation [44] have been used in modelling the diffusion process with a proper applicability, as shown in Equations (1) and (2) below. Focusing on the rejuvenating process with rejuvenator, diffusion coefficient could account a big part to enhance the penetration rate according to Equation (1). Meanwhile, Equation (2) explains the relative factors influencing the diffusion rate, which illustrates that a smaller viscosity and molecular size increases the diffusion coefficient generally. PR thereby engendered more efficient rate of penetration in aged asphalt because of a smaller viscosity, according to the previous analysis.

(1) $$J=-D*\frac{\partial c}{\partial x}.$$

where, *J* is diffusion flux (mol/m^2^·s), c is concentration (mol/m^3^), *x* is the diffusion depth (m), and *D* is the diffusion coefficient.

(2) $$D=\frac{k_{B}T}{6\pi \mu \left(R\right)}$$

where, *D* is diffusion rate (m^2^/s), *R* is the mean molecular radius, *μ* is dynamic viscosity (Pa·s), *k_B_* is Boltzmann’s constant (1.3807 × 10^−23^ J/K), and *T* is absolute temperature (K).

#### 3.2.2. Penetration Principle for Interface

Adding mixed penetrants was an essential procedure to abate the molecular surface energy of rejuvenator as preparing PR, aiming to decrease the interface contact angle and then promote the penetration rate into aged asphalt [45].

**Figure 7:**
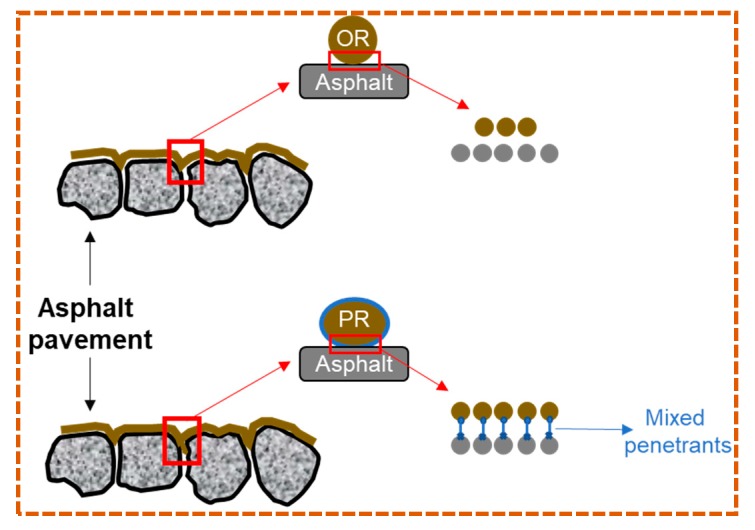
Improving penetration rate with mixed penetrants.

Figure 7 depicts the mechanisms of improving PR penetration rate with mixed penetrants. Theoretically, aged asphalt is a highly polar polymer material and has a complex molecular composition, while rejuvenator is a non-polar oil component, so sufficient contact could be a precondition in efficient penetration for the rejuvenator. Because of mixed penetrants decreasing the contacting angle, more PR molecular can gather in the interface, diffuse and then rejuvenate the aged asphalt. The viscosity is defined as the resistance of fluid during flowing [46]. 

In Figure 7, PR had a smaller viscosity with the addition of penetrants than OR, which allowed to penetrate into the asphalt more quickly. Hence, the viscosity and contact angle in Table 4 demonstrated the more efficient penetrating rate of PR.

### 3.3. Physical and Rheological Performance

#### 3.3.1. Physical Properties

**Figure 8:**
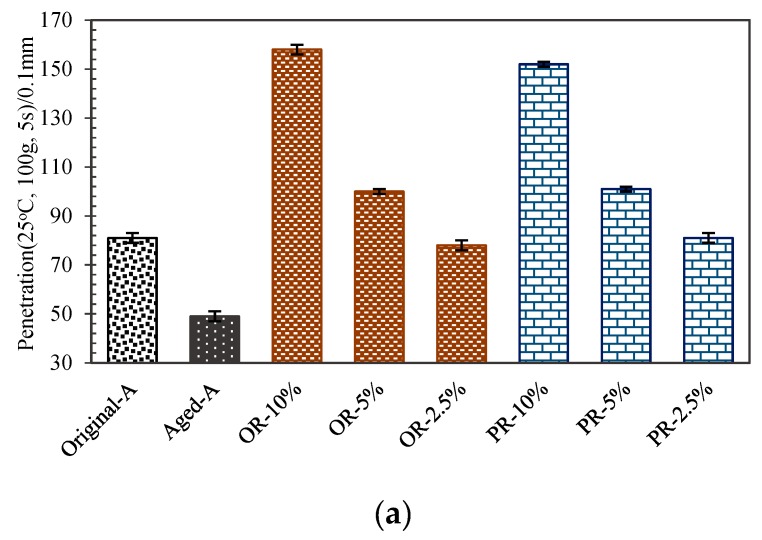

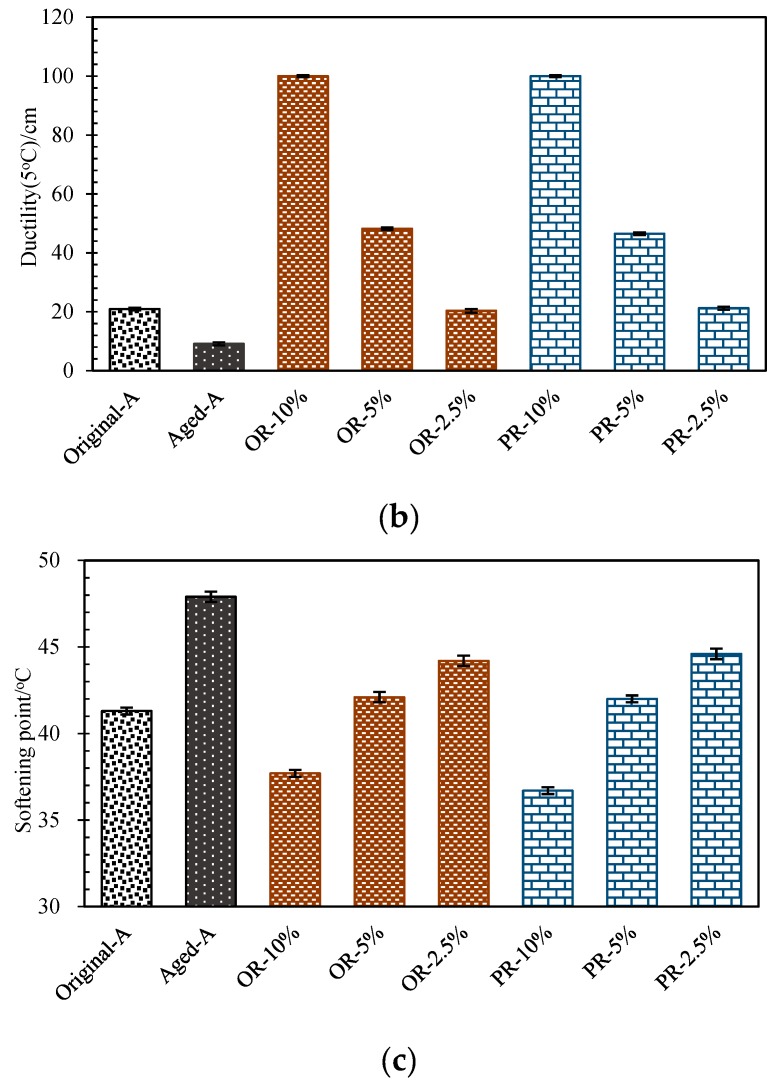
The physical properties of samples: (**a**) penetration; (**b**) ductility; (**c**) softening point.

Asphalt and rejuvenators age to a certain extent during sample heating [47]. Consequently, Original-A was used as a benchmark to decide optimum dosage of rejuvenators. Figure 8 depicts the results of penetration test, ductility test, and softening-point test. It is obvious that both penetration and ductility testing data have increased, while softening point decreased in varying degrees, after PR or OR is added to the aged asphalt.

Meanwhile, OA-2.5% and PA-2.5% have a similar value as Original-A in penetration and ductility, but a minimal increase in soften point. Compared to the aged asphalt sample, a higher data in ductility shows a good cracking resistance at low temperature, and penetration results indicates a good shear resistance because of the rejuvenators. It also demonstrated that the rejuvenators have a positive efficiency in softening the aged asphalt, but excessive dosage will lead to aged asphalt being too soft to handle stress. Moreover, the subtle differences of OA-2.5% and PA-2.5% softening points reflected a better stability than other dosages in high temperature environment. Comprehensively, it can be concluded that 2.5% weight of rejuvenator could result in the most effective regeneration performance on aged asphalt, according to these physical test results.

Figure 8 also shows that the same dosage of OA and PA had same effect on the physical properties of aged asphalt. This phenomenon indicated the penetrant had no effect on the physical properties of the aged asphalt, only accelerated the diffusion rate of the PA in the aged asphalt, when the rejuvenators were added to the aged asphalt by agitation.

#### 3.3.2. High-Temperature Rheological Properties

**Figure 9:**
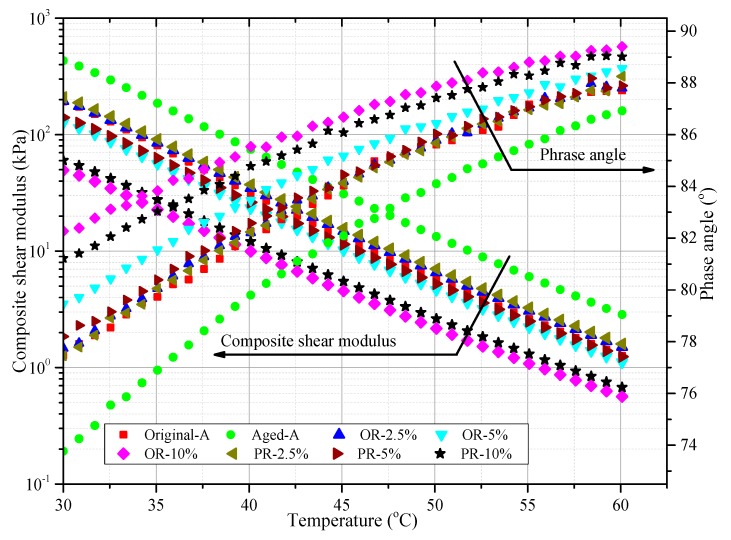
Changes in phase angles and composite shearing modulus because of rejuvenators.

The changes of phase angles and composite shearing modulus because of rejuvenators are shown in Figure 9. Within 30–60 °C, the G* value was found to decrease with higher temperature. Because the movement intensification of asphalt molecules resulted in molecular cross-linking and weak molecular force as the temperature increases. Aged-A had the highest G*, then OA-2.5% and PA-2.5% had a similar G* with Original-A in the temperature range, which illustrated that adding rejuvenators could recover the viscosity of aging asphalt partially. But excessive dosage of rejuvenator might cause worse stiffness modulus and more rutting possibility of asphalt pavements in summer. Phase angle of samples also indicated a similar trend that OA-2.5% and PA-2.5% were closer to Original-A, and the Aged-A had the smallest phase angle, which reflected that the aging program of asphalt would enhance its elasticity. The viscosity of aged asphalt can be partially recovered by both rejuvenators.

**Figure 10:**
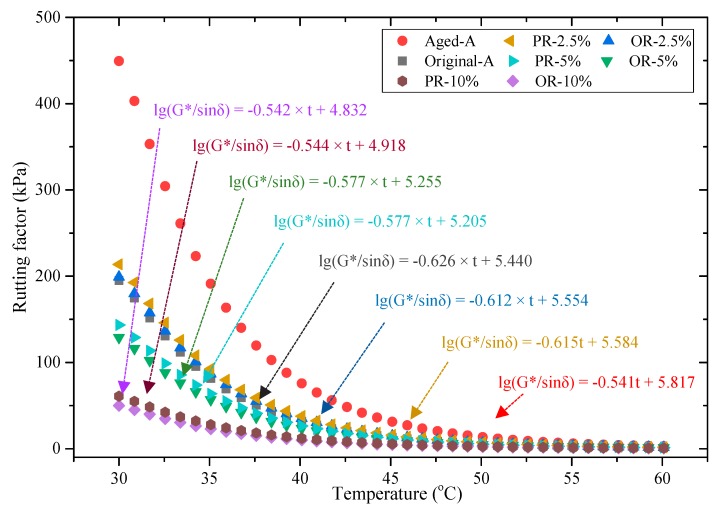
Regeneration effect on rutting factors.

Meanwhile, the regeneration effects on rutting factors are illustrated in Figure 10. Aged-A had the best rutting resistance for its small viscosity. It can be also found that lower rutting factor was obtained with increasing rejuvenators usage, such as PA-10% and OA-10%. Because of the rejuvenators added, aged asphalt became softened and easy to deform at high temperature resulting in rutting with multiple periodic loads, which corresponds to other research results such as reference [48]. Hence, 2.5% rejuvenators of mass percentage of asphalt should be the optimal dosage.

DSR [49] tests composite shear modulus (G*) and phase angle (δ) within 30–60 °C. Then the Equation (3), linear regression equation for the common logarithm of rutting factor (G*/sinδ) and temperature, was used to calculate the absolute value of the slope, which was named modulus temperature susceptibility (GTS) [50]. Afterward, analyzing the GTS can obtain the deforming rate of asphalt during temperature rising. Generally, samples show stronger temperature sensitivity as GTS value increases [51].

(3) $$\mathrm{lg}\frac{G^{*}}{\sin\delta }=-\mathrm{GTS}\cdot t+K_{1}$$

where, G*/sinδ is the rutting factor, kPa; t is temperature, °C; K_1_ is regression constant; GTS is the absolute value of the slope.

Table 5 shows the GTS values, regression constant, and square of correlation coefficient in regression equation. The minimum GTS appeared at aged asphalt sample, while maximum at original asphalt sample. It reflected that temperature sensitivity of the asphalt became weaker after aging. Moreover, 10 wt% usage rejuvenators (both PR and OR) got similar GTS values with aged asphalt. It was explained that exceeding stiffness of aged asphalt obstructed its deformation. On the contrary, the addition of 10 wt% rejuvenator caused over softening and deformation of the samples evidently as the temperature changes. Therefore, excessive rejuvenator would lead to a worse stiffness modulus and more rutting possibility. However, 2.5 wt% samples had a close value with the original sample. It signaled that appropriate usage of rejuvenator can restore the temperature sensitivity of aged asphalt to a certain extent, resulting in the restoration of the pavement service performance gradually.

#### 3.3.3. Low-Temperature Rheological Properties

BBR [52] tests creep stiffness (S) and creep rate (m-value) of asphalt binder. Creep stiffness modulus indicates the ability of asphalt mixture to resist permanent deformation. Creep rate indicates the stress relaxation ability of asphalt at low temperature. Samples reflects a better low temperature crack resistance as S being smaller and m-value being higher. The asphalt beams were tested at −12 °C in this research.

**Figure 11:**
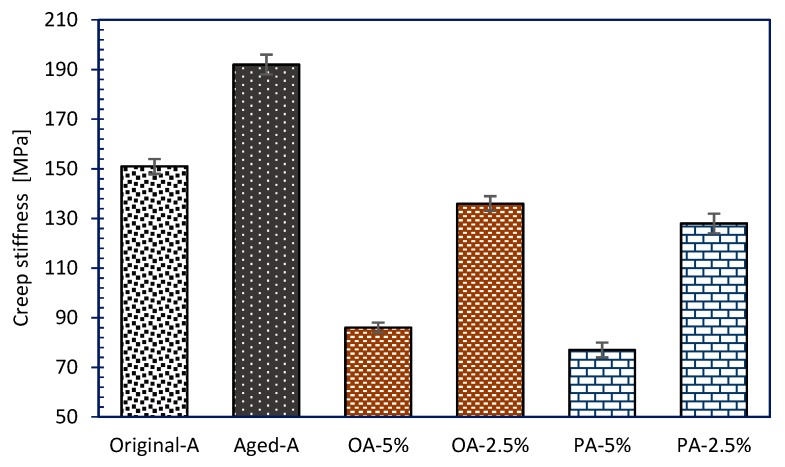
Creep stiffness (S) of asphalt binders with different rejuvenators.

**Figure 12:**
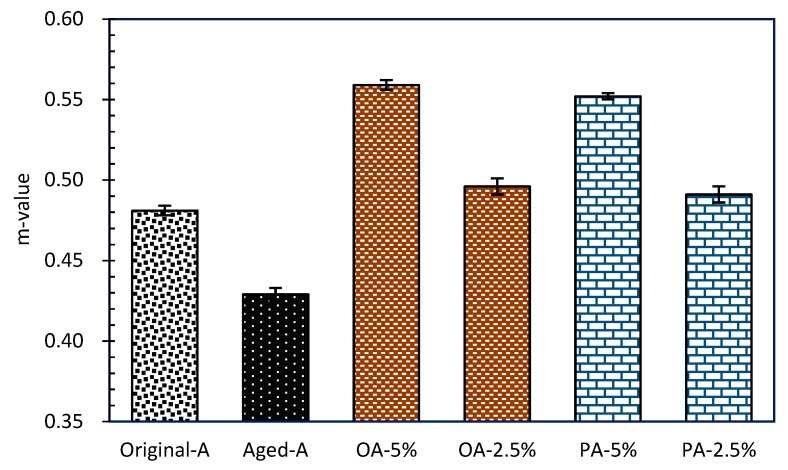
Creep rates (m-value) of asphalt binders with different rejuvenators.

According to the Strategic Highway Research Program, stiffness modulus of asphalt samples is ought to be smaller than 300 MPa at measuring temperature, besides whose creep rate should be greater than 0.300 [53,54]. Figure 11 and Figure 12 showed the BBR test results. Since 10 wt% usage rejuvenators (OA-10% and PA-10%) caused over softening of the asphalt beams, the excess creep rate which was beyond the test range cannot be recorded then. From the creep stiffness data in Figure 11, it can be demonstrated that aged asphalt has the biggest S, nearly 192 MPa. After that, S decreased gradually with increasing rejuvenators dosages. That means that both OR and PR could increase the viscosity of the aged asphalt, improving the low temperature crack resistance effectively. The similar law is shown for m-value in Figure 12. Increasing the adding dosages got a bigger m-value which indicated that rejuvenators could soften and increase the creep rate of the aged asphalt. Moreover, the dosage of 2.5% rejuvenators in aged asphalt showed better crack resistance than original asphalt. Aged asphalt sample showed the smallest m-value, because low temperature obstructed its deformation, indicating its worse temperature sensitivity at cold condition.

## 4. Conclusions

This study proposed three test methods to evaluate the diffusion characteristics of rejuvenator in aged asphalt binder. A feasible rejuvenator with good permeability was designed as well by using emulsified asphalt and waste cooking oil. Both regenerative performance and permeability of OR and PR have been investigated. The following conclusions can be obtained.

(1) The applied penetrants can reduce the contact angle between the liquid rejuvenator and the aged asphalt binder, because of the diffusion mechanism and its interface information. It improved the efficiency of diffusion and permeability between rejuvenators and aged asphalt binder.
(2) The self-designed sinking time test, softening rate test, and gravitational collapsing test had been approved as effective ways to compare the diffusion efficiency between different rejuvenators. All these three tests agreed with each other quite well. PR has a better permeability into aged asphalt than that of OR. It has shorter sinking time, softening time, and collapsing time.
(3) Rejuvenators increased the penetration and ductility values, while decreased the softening point. The addition of 2.5 wt% rejuvenator could result in the most effective regeneration performance on aged asphalt, according to both physical and rheological tests results.
(4) Both modulus and phase angle indicated that adding rejuvenators can recover viscosity of aging asphalt partially. Moreover, BBR test demonstrated that the addition of rejuvenators can restore the rheology and temperature sensitivity of aged asphalt in cold condition. But excessive dosage of rejuvenator may cause a worse stiffness modulus, increasing the rutting possibility.

## Figures and Tables

**Table 1:** Fundamental properties of AH 90 asphalt binder.

Items	Parameter	Results
Physical properties	25 °C Penetration (0.1 mm)	80.5
	10 °C Ductility (cm)	>100
	Softening point [°C]	41.3
	135 °C Viscosity (Pa·s)	0.533
Chemical compositions	Saturates (%)	15.7
	Aromatics (%)	31.3
	Resins (%)	41.8
	Asphaltenes (%)	11.2

**Table 2:** Fundamental properties of waste cooking oil (WCO).

Items	Parameter	Results
Physical properties	pH values	4.2
	Density (g/mL)	0.920
	25 °C Viscosity (cP)	57.0
Chemical compositions	Saturates (%)	26.5
	Aromatics (%)	28.1
	Resins (%)	45.4
	Asphaltenes (%)	N/A

**Table 3:** Labels for rejuvenated binders.

Rejuvenators and Dosages	Oil Rejuvenator			Penetrative Rejuvenator		
	10%	5%	2.5%	10%	5%	2.5%
Labels	OA-10%	OA-5%	OA-2.5%	PA-10%	PA-5%	PA-2.5%

**Table 4:** The results of permeability tests for PR and OR.

Rejuvenators	25 °C Viscosity (Pa·s)	Contact Angle (°)		Sinking Time (s)	Softening Time (s)	Collapsing Time (s)
		Aged Asphalt	Original Asphalt			
OR	1.278	53.1	51.6	2660	1974	2873
PR	0.478	34.8	34.6	1557	1356	1926

**Table 5:** The regression equation for modulus temperature susceptibility (GTS) value.

Samples	GTS	K_1_	R^2^
Original-A	0.626	5.440	0.998
Aged-A	0.541	5.817	0.999
OA-10%	0.542	4.832	0.998
OA-5%	0.577	5.255	0.998
OA-2.5%	0.612	5.554	0.998
PA-10%	0.544	4.918	0.998
PA-5%	0.577	5.205	0.998
PA-2.5%	0.615	5.584	0.998
